# Supplementary material for: Impact of vaccination on infection or death from COVID-19 in individuals with laboratory-confirmed cases: Case-control study
Source: PLoS One. 2023 Aug 3;18(8):e0265698. doi: 10.1371/journal.pone.0265698 (PMC10399771; doi:10.1371/journal.pone.0265698)
Supplement: Attachment — Submitted filename: Answers.docx [file pone.0265698.s002.docx]

**“Impact of vaccination on infection or death from COVID-19 in individuals with laboratory-confirmed cases: case-control study”**

**General.**

**1. Please ensure that your manuscript meets PLOS ONE's style requirements, including those for file naming.**

A = We did the changes.

**2. "In your ethics statement in the manuscript and in the online submission form, please provide additional information about the patient records used in your retrospective study. Specifically, please ensure that you have discussed whether all data were fully anonymized before you accessed them.**

A = We included the Ethical approval. Also, de basis was fully anonymized.

**3. We note that you have stated that you will provide repository information for your data at acceptance. Should your manuscript be accepted for publication, we will hold it until you provide the relevant accession numbers or DOIs necessary to access your data. If you wish to make changes to your Data Availability statement, please describe these changes in your cover letter and we will update your Data Availability statement to reflect the information you provide.**

A = Available on figshare: 10.6084/m9.figshare.21691229

**4. Please include captions for your Supporting Information files at the end of your manuscript, and update any in-text citations to match accordingly.**

A = We did the changes.

**5. Please include a copy of Tables 1 to 5 which you refer to in your manuscript.**

A = the tables were loaded, as well as the supporting information tables.

**Review Comments to the Author.**

**Reviewer #1: The goal of this study is relevant. Although that are a few points that require attention and better discussion.**

**1. Better graphic representation of the results is needed.**

A = Graphics files have been modified.

**2. The time after vaccination should be taken into consideration since studies have shown that this could be a limiting factor for protection. Thus, comparing people vaccinated in** **different moments could be a problem.**

A = It was omitted to include in the operational definition that the time is from the second dose or from the only dose, depending on the biologic. The timing of vaccination was not possible to control. The strategy in Mexico was to start by older adults to younger.

**3. The fact that the vaccine source was self-reported makes the conclusions weaker. Is possible to confirm the source of vaccination?**

A = The information was obtained from an already structured information system, so it was not possible to analyze other variables of interest. In Reviewer 2's comment on limitations, the statement was modified to address both comments.

**4. The differential bias between cases and controls should be taken into consideration and better discussed in the manuscript.**

A = The cases and controls were taken from the same population with initial respiratory symptoms; however, the controls were negative in the PCR tests, so the answers to the questioning had the same opportunity to be identified.

**5. The figure need to have better quality and higher resolution.**

A = Graphics files have been modified.

**Reviewer #2: Article with a very current theme, but needs adjustments for publication in PLOS ONE.**

**1. ABSTRACT / Methods – “... those with a positive result were considered cases, and 60 those with a negative result were considered controls.” - What is the ratio between cases and controls? This information needs to be detailed in the summary.**

A = The ratio between cases and controls was 1:1.3.

**2. ABSTRACT / Results – “of whom 40,192 were considered 65 cases and 54,224 controls;” - Would the 1:1 ratio be able to identify the differences? Explain.**

A = The ratio between cases and controls was 1:1.3.

**3. “3,781 (4.00%) had been vaccinated against COVID-19.” - What is the proportion of vaccination in cases and controls? Explain.**

A = The vaccination proportion in cases was 3.35% and controls 5.31%. The low vaccination coverage is due to the fact that the study was carried out when the application of vaccines was just beginning. However, a lower proportion of vaccinated cases is observed.

**4. “(OR = 76; 95% CI 69 0.66-0.87).” - OR = 76 – Review this information.**

A = With the application of the vaccine, there was a protective effect against mortality (OR = 0.76; 95% CI 0.66-0.87).

**5. METHODS. Operational definitions -> “...at least 15 days before the onset of symptoms” - From the second dose? From the first dose? It would impact.**

A = From the second dose.

**6. RESULTS. It is important for the table to show that the groups are homogeneous, but characteristic. Otherwise (heterogeneous), there could be a bias.**

A = The study included the universe of work technically to avoid any type of selection bias and logistic regression was performed to control possible confounders.

**7. DISCUSSION. It is well structured, however very directed to the basic field of science. Restructuring to respond to the objective from a public health perspective: What was the impact of vaccination on population health? I suggest better highlighting the benefits of vaccines for the health of the population.**

A = It is now known from published studies that counties/countries with higher vaccination coverage had fewer COVID-19 cases and deaths per head of population and the measured effectiveness in counties/countries with high vaccine coverage was reassuringly large and the vaccination benefits whole communities, and indeed it does when coverage is high. Vaccine rollout began in Mexico with older and otherwise vulnerable people and progressively included younger and less vulnerable people; although we did not investigate the effect of vaccination by age, but our findings of this study also make clear that many more lives could have been saved, and will be saved. That’s why we added: “Although it is already known that vaccination against COVID-19 reveals that our findings of this study also make clear that many more lives could have been saved, and will be saved;^42^ not only for the population to know that information; but also for the health authorities when vaccination had only a mild effect on the substantial reduction in transmission rate and mortality/fatality rates when vaccination coverage are low or delayed.^43^”

**8. What are the limitations of the study? Explain better.**

A = The limitations of the study are described in the discussion; however, detailing the statement with that subtitle was omitted. has been added to the manuscript. “The most important limitation was the source of the vaccine was self-reported; however, the data was obtained from an already structured information system, so it was not possible to analyze other variables of interest., and there was differential bias between cases and controls; for example, 1) high-risk groups (people…

Sincerely,

The authors.
