# Supplementary material for: Impact of vaccination on infection or death from COVID-19 in individuals with laboratory-confirmed cases: Case-control study
Source: PLoS One. 2023 Aug 3;18(8):e0265698. doi: 10.1371/journal.pone.0265698 (PMC10399771; doi:10.1371/journal.pone.0265698)
Supplement: Attachment — Submitted filename: Answers.docx [file pone.0265698.s003.docx]

**ANSWERS.**

**Comments to the Author**

1. If the authors have adequately addressed your comments raised in a previous round of review and you feel that this manuscript is now acceptable for publication, you may indicate that here to bypass the “Comments to the Author” section, enter your conflict of interest statement in the “Confidential to Editor” section, and submit your "Accept" recommendation.

Reviewer #1: All comments have been addressed

Reviewer #2: All comments have been addressed

2. Is the manuscript technically sound, and do the data support the conclusions?

The manuscript must describe a technically sound piece of scientific research with data that supports the conclusions. Experiments must have been conducted rigorously, with appropriate controls, replication, and sample sizes. The conclusions must be drawn appropriately based on the data presented.

Reviewer #1: Partly

Reviewer #2: Yes

3. Has the statistical analysis been performed appropriately and rigorously?

Reviewer #1: Yes

Reviewer #2: Yes

4. Have the authors made all data underlying the findings in their manuscript fully available?

The PLOS Data policy requires authors to make all data underlying the findings described in their manuscript fully available without restriction, with rare exception (please refer to the Data Availability Statement in the manuscript PDF file). The data should be provided as part of the manuscript or its supporting information, or deposited to a public repository. For example, in addition to summary statistics, the data points behind means, medians and variance measures should be available. If there are restrictions on publicly sharing data—e.g. participant privacy or use of data from a third party—those must be specified.

Reviewer #1: Yes

Reviewer #2: Yes

5. Is the manuscript presented in an intelligible fashion and written in standard English?

PLOS ONE does not copyedit accepted manuscripts, so the language in submitted articles must be clear, correct, and unambiguous. Any typographical or grammatical errors should be corrected at revision, so please note any specific errors here.

Reviewer #1: Yes

Reviewer #2: Yes

**6. Review Comments to the Author**

Please use the space provided to explain your answers to the questions above. You may also include additional comments for the author, including concerns about dual publication, research ethics, or publication ethics.

**Reviewer #1:** Although the manuscript reports important data about a relevant topic, some points should still be considered.

**1. Regarding the time after vaccination, although it was impossible to control, it should be considered in the analysis.**

A = Table 1. “Deaths and survivors, main characteristics”. We added “Onset after vaccination*”

COVID-19 cases had a median number of fewer days after being vaccinated compared to controls 20 vs 42, p<0.000, while in deceased and survivors of COVID-19 there was no statistically significant difference 19 vs 20, p= 0.4181.

**2. The fact that the vaccine source was self-reported compromises the conclusions. Is that possible to access a governmental system to check that? Is that available in Mexico?**

A = Public information is numerical reports. Although the available database is counted, but it is impossible to identify the people notified in the epidemiological surveillance system.

**3. In Methods: when the authors mention that "at least 15 days before the onset of symptoms", the information that it is from the second dose should be added.**

A = This observation was added to the methodology.

**4. The discussion should still be better restructured, considering the importance of vaccination to the population.**

A = It has been modified and paragraphs have been added to the discussion.

**Reviewer #2: Dear Editor and Authors**,

Thank you for the opportunity to review this manuscript. I am grateful for the invitation.

After careful consideration, I feel that the manuscript has a satisfying result and explores a very important topic, but there few weaknesses in the Introduction, methods and discussion:

**The introduction is very concise. I suggest better exploring epidemiological data, including recent international studies, as well as inserting a more robust justification for the study.**

A = The introduction was modified, in order to strengthen the justification of the study.

**The methods are well described, with rigor in data analysis.**

A = Thanks for the observation.

**Explain the period of data collection in the system.**

A = The source of information was the epidemiological surveillance system of COVID-19; therefore, the time of registration of each person was when the medical unit notified the case. For the data collection, for the purposes of the study it was one month later (July 22, 2021).

**In the discussion and conclusions: include how the results of the study can be extrapolated, in order to explain the relevance of this paper.**

A = It has been modified and paragraphs have been added to the discussion.

Kind regards,

The authors.
